# NO-cGMP-K^+^ Channels Pathways Participate in the Antihypertensive Effects of *Attalea phalerata* Martius ex Spreng Oil-Loaded Nanocapsules

**DOI:** 10.3390/pharmaceutics16070842

**Published:** 2024-06-21

**Authors:** Maria Medina de Azevedo, Francislaine Aparecida dos Reis Lívero, Sílvia Beatriz Bürger Tinelli, Jacenir Vieira da Silva, Danielle Ayr Tavares de Almeida, Marco Antonio Utrera Martines, Ariadna Lafourcade Prada, Jesús Rafael Rodríguez Amado, Arquimedes Gasparotto Junior

**Affiliations:** 1 Laboratory of Cardiovascular Pharmacology (LaFaC), Faculty of Health Sciences, Federal University of Grande Dourados (UFGD), Dourados 79804-970, MS, Brazil; mariamedinaazevedo@outlook.com (M.M.d.A.); jacenirsilva@ufgd.edu.br (J.V.d.S.); danielleayr@gmail.com (D.A.T.d.A.); jesusamado@ufgd.edu.br (J.R.R.A.); 2 Laboratory of Cardiometabolic Pharmacology, Department of Pharmacology, Federal University of Parana (UFPR), Curitiba 80060-000, PR, Brazil; francislaine@ufpr.br; 3 Postgraduate Program in Biotechnology Applied to Agriculture, Paranaense University, Umuarama 87502-210, PR, Brazil; silvia.burger@edu.unipar.br; 4 Laboratory of Nanostructured Materials, Metallodrugs and Medicines, Institute of Chemistry, Federal de University of Mato Grosso do Sul, Campo Grande 79070-900, MS, Brazil; marco.martines@ufms.br; 5 Postgraduate Program in Biotechnology, Faculty of Pharmacy, Food, and Nutrition, Federal University of Mato Grosso do Sul, Campo Grande 79070-900, MS, Brazil; lafourcade.ariadna@ufms.br

**Keywords:** Arecaceae, acurí, cardiovascular, drug carrier, hypertension, nanoformulation

## Abstract

*Attalea phalerata* Martius ex Spreng is a palm tree that is widely distributed in the Central-West region of Brazil. In this study, we investigated whether the oil-loaded nanocapsules of *A. phalerata* (APON) have acute and long-lasting antihypertensive effects in male spontaneously hypertensive rats (SHR), as well as explored the underlying molecular mechanisms. APON was prepared using the interfacial polymer deposition method. The particle size, polydispersity index, and zeta potential were investigated using dynamic and electrophoretic light scattering. The antihypertensive effects of APON (administered at doses of 1, 3, and 10 mg/kg) were evaluated after acute intraduodenal administration and after 7 days of oral treatment. To investigate the molecular pathways involved, we used pharmacological antagonists and inhibitors that target prostaglandin/cyclic adenosine monophosphate, nitric oxide/cyclic guanosine monophosphate, and potassium channels. Both acute and prolonged administration of APON (at doses of 3 and 10 mg/kg) resulted in a significant reduction in systolic, diastolic, and mean arterial pressure. Prior treatment with a non-selective nitric oxide synthase inhibitor (Nω-nitro-L-arginine methyl ester), guanylyl cyclase inhibitor (methylene blue), or non-selective calcium-sensitive K^+^ channel blocker (tetraethylammonium) abolished the antihypertensive effects of APON. Our study showed that *A. phalerata* oil-loaded nanocapsules have a significant antihypertensive effect in SHR after both short-term and long-term (7-day) use. This effect seems to rely on the vascular endothelium function and involves the NO-cGMP-K^+^ channel pathway. This research suggests a new direction for future studies to definitively prove the therapeutic benefits of APON in treating cardiovascular disease.

## 1. Introduction

Cardiovascular disease (CVD) remains a global health challenge, significantly contributing to mortality and morbidity rates worldwide. Hypertension, a prevalent CVD, significantly increases the risk of heart failure, coronary artery disease, and chronic kidney disease [1,2]. Traditional populations have a longstanding practice of using medicinal plants and herbs in the management of cardiovascular ailments, particularly hypertension. These natural products contain a rich source of biologically active compounds, favoring the development of new herbal medicines [3,4]. Despite the rich phytochemical spectrum, many plant-based preparations have low solubility and gastrointestinal absorption [5]. Therefore, the use of nanocarriers can promote the development of innovative formulations from natural products, becoming effective pharmaceutical forms for the treatment of cardiovascular diseases [6].

Palm tree oils are commonly used as food and remedies by traditional communities [6]. The palm tree oil (dendé oil) obtained from the *Elaeis guineensis* and Coconut oil (from *Cocos nucifera*) are the palm tree oils most known and commercialized around the world. However, there is a diversity of oils produced by different species of the Arecaceae family which are little known but widely used by native populations both as food and as remedy. Attalea phalerata Martius ex Spreng, commonly known as acurí, is a palm tree native to Central-West Brazil, and particularly, to the Cerrado biome [7,8]. Traditionally, indigenous populations have used the kernel oil of *A. phalerata* as a substitute for palm and coconut oil in cooking [6,7]. Additionally, acurí oil has been used in traditional medicine to treat fever, skin lesions, respiratory problems, and inflammation [7,9].

Recent research suggests potential health benefits beyond its traditional uses. Acácio et al. [8] reported that APO contains 79.60% saturated fatty acids (C_8:0_, 8.73%; C_10:0_, 7.91%; C_12:0_, 43.33%; C_14:0_, 10.80%; C_16:0_, 6.69%; C_18:0_, 2.20%) and 20.35% unsaturated fatty acids (C_18:1n9c_, 18.01% and C_18:2n6c_, 2.35%). Dodecanoic acid (C_12:0_), also known as lauric acid is the prevalent saturated fatty acid present in APO. It has diverse beneficial effects, including cholesterol reduction [8]. The prevalent unsaturated fatty acid contained in APO is 9-octadecenoic acid (C_18:1n9c_, also known as oleic acid). Oleic acid reduces total cholesterol and low-density lipoprotein cholesterol and contributes to regulating the blood pressure. However, APO was never evaluated in order to validate its utility in those health conditions.

Our research group has developed a formulation called acurí oil-loaded nanocapsules (APON) that demonstrates anti-inflammatory and cytotoxic effects against cancer cells [8]. APON reduces serum triglycerides and total cholesterol in rats, suggesting potential applications in preventing and treating cardiovascular diseases [8]. Additionally, the study found APON to be non-toxic at high doses in rats, with an LD_50_ exceeding 2000 mg/kg [8].

This study investigated the effects of *Attalea phalerata* Martius ex Spreng oil-loaded nanocapsules (APON) on blood pressure in male spontaneously hypertensive rats (SHR). We specifically assessed whether APON administration resulted in acute and/or long-term antihypertensive effects. Additionally, we explored the underlying molecular mechanisms responsible for the observed hemodynamic changes.

## 2. Materials and Methods

### 2.1. Chemicals

Heparin was obtained from Hipolabor (Belo Horizonte, MG, Brazil). Xylazine and ketamine hydrochloride were sourced from Syntec in São Paulo, SP, Brazil. Methylene blue, 2′,5′-dideoxyadenosine (DDA), indomethacin, tetraethylammonium (TEA), and Nω-nitro-L-arginine methyl ester (L-NAME) were obtained from Sigma-Aldrich (St. Louis, MO, USA). Kollicoat^®^ MAE 100P was kindly supplied by BASF, São Paulo, Brazil. Tween 80 was purchased from Crodapharma, São Paulo, (Brazil).

### 2.2. Plant Material

Ripe fruits of *A. phalerata* were harvested in the municipality of Jaraguari, Mato Grosso do Sul, Brazil (20°6′58″ S 54°25′44″ W). The Department of Botany at the Federal University of Mato Grosso do Sul confirmed the botanical identification of the species.

### 2.3. Oil Extraction

Ripe fruits were air-dried at room temperature for 7 days. Subsequently, the kernels were separated from the fruit and ground using an electric grinder. The extraction was performed using 200 g of the grinding fruit kernel in 1000 mL of n-hexane. The mixture was placed in the dark for 7 days and stirred thrice a day. The n-hexane liquid holding the oil was filtered through filter paper to remove physical impurities and transferred to a rotary evaporator under vacuum at a temperature of 50 °C until complete solvent removal. The oil was stored in an amber bottle at room temperature until further use [8].

### 2.4. Physicochemical Characterization

The organoleptic properties, such as color, aroma, and appearance of the APO, were documented. The physiochemical properties, including refractive index, relative density, acidity index, iodine value, and saponification value, were evaluated in accordance with the Brazilian Pharmacopeia [8,9,10].

### 2.5. Gas Chromatography/Mass Spectroscopic Analysis

The fatty acid profile of the APO was evaluated following the methodology developed by Acácio et al. [8]. Gas Chromatography coupled to Mass Spectrometry (Shimadzu, model GC-2010, Kyoto, Japan) was used with a Flame Ionization Detector (FID) and a split/splitless injector. The separation was carried out on a 30 m fused silica capillary column with a diameter of 0.25 mm, BPX-70 (70% Cyanopropyl polysilphenylenesiloxane) from Sigma Aldrich, USA. The operating parameters were as follows: detector temperature of 250 °C, injector temperature of 250 °C. The initial column temperature was set at 80°C (3 min), gradually increased to 140 °C at a rate of 10 °C/min, further increased to 240 °C at a rate of 5 °C/min, and held at that temperature for 11 min. Helium (from White Martins, Brazil) was used as the carrier gas with a flow rate of 1.0 mL/min, synthetic air and hydrogen as the detector gas, and nitrogen as the auxiliary gas (make-up gas). The injection volume was 1μL. Identification and quantification of fatty acids were performed by comparing the retention time of fatty acid methyl esters in the sample with that of the standard (FAME mix, 100 mg—37 components). The quantitation was expressed as a percentage of the total found fatty acids.

The analytical methodology was developed and validated for this purpose. The method’s recovery (accuracy) was 100 ± 2.11%, the precision was ± 1.12%, the limit of detection (LOD) was 2.50 ppm, and the limit of quantification (LOQ) was 8 ppm. The mean and the standard deviation of three replicates were reported.

### 2.6. Nanocapsules Preparation

The Acurí oil-loaded nanocapsules were synthesized using the nanoprecipitation method [11] with some modifications [8]. For the preparation of the organic phase, 1 g of Kollicoat^®^ MAE 100P was dissolved in 20 mL of a mixture of absolute ethanol and acetone (3:1). The mixture was stirred at 400 rpm for 7 min. Then, 0.5 g of Span80 and 0.5 g of Acurí oil were added, with the stirring kept at 400 rpm for 10 more min. The aqueous phase consisted of 100 mL of Milli-Q water and 0.5 g of the non-ionic surfactant Tween 80. The organic phase was added dropwise to the aqueous phase with agitation (650 rpm) and stirred for 15 min. The mixture was sonicated using an ultrasonic probe (Sonics Vibra-Cell VCX 750, Sao Paulo, Brazil) with a power of 8.0 W for 7 min. Then, the solvent mixture was evaporated on a heating plate at 50 °C with magnetic stirring at 650 rpm for 24 h. The nanocapsule suspension was transferred to an amber vial; the volume was adjusted to 100 mL with Milli-Q water, and it was stored at 25 ± 2 °C for 24 h prior to characterization [8].

### 2.7. Nanocapsules Characterization

#### 2.7.1. Particle Size and Polydispersity Index

The particle size and homogeneity (polydispersity index) were assessed using Photon Correlation Spectroscopy with a Zetasizer Nano ZS instrument (Malvern, UK). The measurements were conducted at a laser wavelength of 633 nm, scattering angle of 173°, and temperature of 25 °C. Three measurements were taken for each sample, and the mean ± standard deviation was reported [8,11].

#### 2.7.2. ζ-Potential

The ζ-potential was measured using Electrophoretic Light Scattering in a Zetasizer Nano ZS (Malvern, UK) with gold electrode polycarbonate cuvettes (DTS1060, UK). The measurements were conducted at 25 °C with a voltage of 150 V. Three replicates were carried out, and the results were reported as mean ± standard deviation [8,11].

#### 2.7.3. pH Evaluation

The pH of the suspension of nanocapsules was measured using a pH meter (Gehaka PG2000). The equipment was calibrated using buffer solutions of pH 4, 7, and 10 before taking the measurements. The measurements were carried out in triplicate.

### 2.8. Animals

Three-month-old male SHR rats, weighing 310–340 g, were obtained from the animal facility at the Federal University of Grande Dourados (UFGD). The rats were housed in a controlled vivarium with a 12-h light/dark cycle, maintained at a temperature of 22 ± 3 °C and a humidity level of 50–60%. They were provided with *ad libitum* access to filtered water and standard food pellets. All animal handling procedures were conducted in accordance with the guidelines approved by the Ethics Committee in Animal Experimentation of UFGD (protocol no. 07/2020).

### 2.9. Investigation of APON Effects on Arterial Pressure and Heart Rate

To evaluate the acute effects of APON, male spontaneously hypertensive rats (SHR) were subjected to continuous anesthesia via inhalation of isoflurane (2 to 3%). Additionally, each rat received a single subcutaneous injection of heparin (50 IU). Subsequently, the left carotid artery was cannulated and connected to a pressure transducer, which was interfaced with the PowerLab data acquisition system utilizing LabChart 8.1.28 software for Windows (ADI Instruments, Castle Hill, Australia) to recording SBP, DBP, MAP, and HR levels. After this procedure, different groups of rats (*n* = 6) received varying doses of APON (1, 3, or 10 mg/kg) or enalapril (5 mg/kg) via intraduodenal administration. The control group was given intraduodenal administration of a vehicle (0.9% saline) at a consistent volume of 100 µL per 100 g body weight. Changes in arterial pressure and heart rate (HR) were monitored for 35 min following the treatments.

To evaluate the blood pressure-lowering effects after extended treatment, distinct groups of rats (*n* = 6 per group) were given oral doses of APON (1, 3, or 10 mg/kg), enalapril (5 mg/kg), or a control (filtered water; 100 µL/100 g body weight) once a day for 7 days. On the 8th day, the rats were anesthetized with continuous inhalation of isoflurane (2–3%) and underwent the same surgical procedure as previously described. Changes in blood pressure and heart rate were monitored for 35 min. Each rat received only one substance being studied during both acute and prolonged treatment stages. At the end of the experiments, euthanasia was carried out by administering an overdose of isoflurane inhalation (30–40%).

### 2.10. Involvement of the Prostaglandin/Cyclic Adenosine Monophosphate, Nitric Oxide/Cyclic Guanosine Monophosphate Pathways, as well as K^+^ Channels, in the APON Antihypertensive Effects

After isoflurane anesthesia (2–3%), the left femoral vein was cannulated in different groups of spontaneously hypertensive rats (*n* = 6/group) and connected to an infusion pump (EFF 311, Insight, Ribeirão Preto, Brazil). Subsequently, L-NAME (a non-selective nitric oxide synthase inhibitor; 7 mg/kg/min) or methylene blue (a guanylyl cyclase inhibitor; 150 nmol/kg/min) was continuously infused, or a single injection of DDA (selective adenylate cyclase inhibitor; 0.1 mg/kg), indomethacin (a nonselective cyclooxygenase inhibitor; 3 mg/kg), or TEA (non-selective K^+^ channel blocker; 400 μmol/kg) was administered intraperitoneally. Afterward, APON (3 mg/kg) was administered via the intraduodenal route, and arterial pressure levels were recorded for 35 min. Each rat received only one of the substances being investigated. The total volume injected into the animals during the infusion period was 1000 μL. At the end of the experiments, all animals were euthanized using an overdose of isoflurane (30–40% by inhalation).

### 2.11. Statistical Analyses

Statistical analyses were performed using one-way analysis of variance (ANOVA) followed by the Bonferroni post hoc test. Results are expressed as mean ± standard error of the mean of 6 animals per group. Statistical significance was set at *p* < 0.05. GraphPad Prism 10 for macOS (San Diego, CA, USA) was used for constructing the graphs and for all statistical analyses.

## 3. Results

### 3.1. Oil Extraction and Characterization

The *Athalea phalerata* oil (APO) is non-viscous and translucent liquid, yellowish, with the aroma of the ripe fruit. The extraction yielded 35.25% (*w*:*w*) of APO. APO exhibited a refractive index (at 30 °C) of 1.4590 ± 0.0105, a density (30 °C) of 0.925 ± 0.040, an acidity value of 0.12 ± 0.02%, an iodine value of 21.50 ± 3.15, and a saponification value of 237.80 ± 10.25.

### 3.2. Fatty Acid Profile

The fatty acid composition of the APO is shown in Table 1. It is important to note that the lipid composition of Acurí oil includes just eight fatty acids, with saturated fatty acids making up 79.60% and the predominant compound being dodecanoic acid. Unsaturated fatty acids make up 20.35%, with the prevalent one being octadecenoic acid.

### 3.3. Nanocapsules Properties

**Figure 1:**
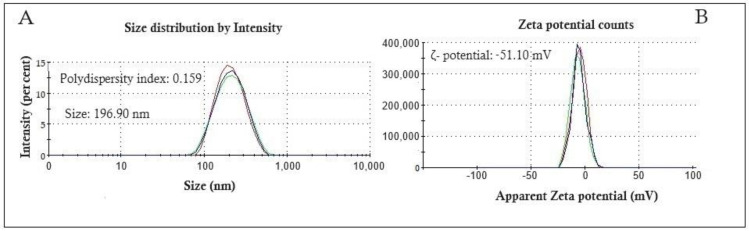
Particle size distribution (**A**) and ζ-potential (**B**) of *A. phalerata* kernel oil-loaded nanocapsules (APON).

The particle size distribution and ζ-potential of APON are shown in Figure 1. Nanocapsules showed a particle size of 196.90 nm (Figure 1A). The particle size distribution graph exhibits a narrow peak base, typical of a system with a low polydispersity index (0.159). Nanocapsules showed a ζ-potential of −59.10 mV (Figure 1B), and a pH of 5.14.

#### Patent

This product was patented as: Jesus Rafael Rodríguez Amado, Ariadna Lafourcade Prada; Bianca Rodrigues Acácio; Marco António Utrera Martines; Renata Trentin Perdomo. Compositions containing oil from acurí fruits (*Scheelea phalerata* (Mart. ex Spreng.) Burret: Pharmacologic, cosmetic, and nutraceutical utilities (in Portuguese). Under deposit number: BR 102022015777-4 A2. Nacional Institute of Industrial Property (INPI), Brazil. Deposit date: 09/08/2022. Publication date: 20/02/2024. Revista da Propriedade Industrial, Rio de Janeiro, Brazil. https://revistas.inpi.gov.br/rpi/ (accessed on 9 May 2024). Section VI, Patents: p. 224.

### 3.4. Antihypertensive Effects of APON in SHR2

**Figure 2:**
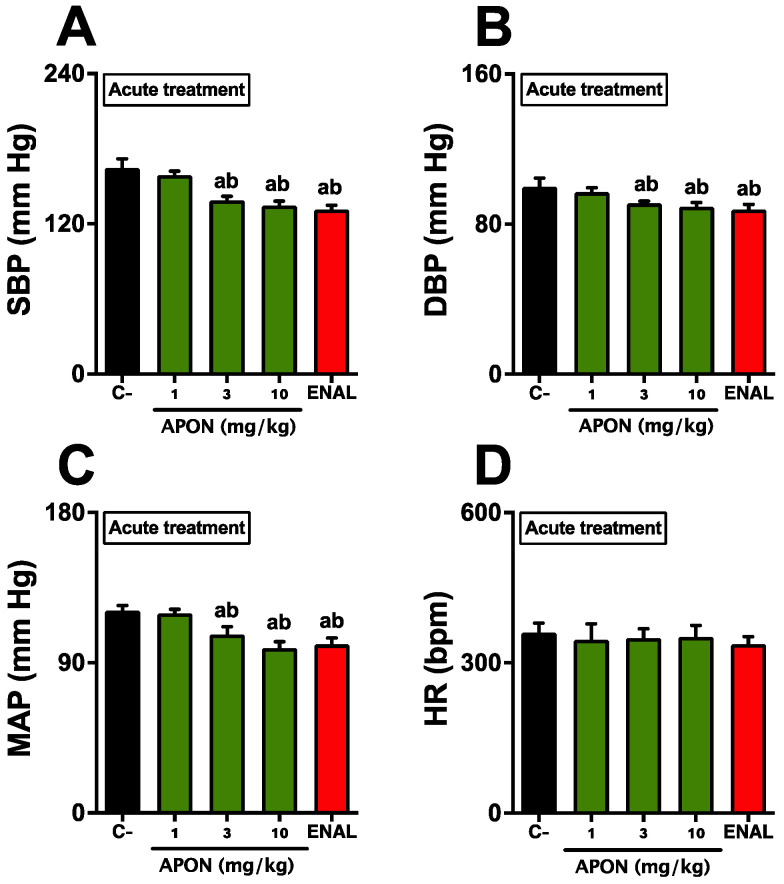
Acute antihypertensive effects of *Attalea phalerata* oil-loaded nanocapsules (APON). APON was administered intraduodenally in anesthetized rats. The systolic blood pressure (SBP; **A**), diastolic blood pressure (DBP; **B**), mean arterial pressure (MAP; **C**), and heart rate (HR; **D**) are displayed. C-, effect of vehicle control (0.9% saline, 200 μL). The results are expressed as mean ± SEM (*n* = 6/group). ^a^ *p* < 0.05 when compared with respective control group; ^b^ *p* < 0.05 when compared with 1 mg/kg APON (ANOVA followed by Bonferroni post hoc test).

After a 15 min stabilization period and before administering any substances, the baseline systolic blood pressure (SBP), the diastolic blood pressure (DBP), and the mean arterial pressure (MAP) of the animals (SHR) were recorded as 161.4 ± 4.2, 99.3 ± 3.9, and 122.5 ± 4.6 mmHg, respectively, while the heart rate (HR) was at 345 ± 39 beats per minute (bpm). Administering intraduodenal APON at doses of 3 and 10 mg/kg resulted in a decrease of approximately 20 mmHg in SBP, DBP, and MAP (Figure 2A–C). There was no noticeable effect on HR with any of the substances given (Figure 2D). Further analysis showed no statistically significant differences in the recorded data following the administration of enalapril (5 mg/kg) compared to receiving APON at doses of 3 and 10 mg/kg.

**Figure 3:**
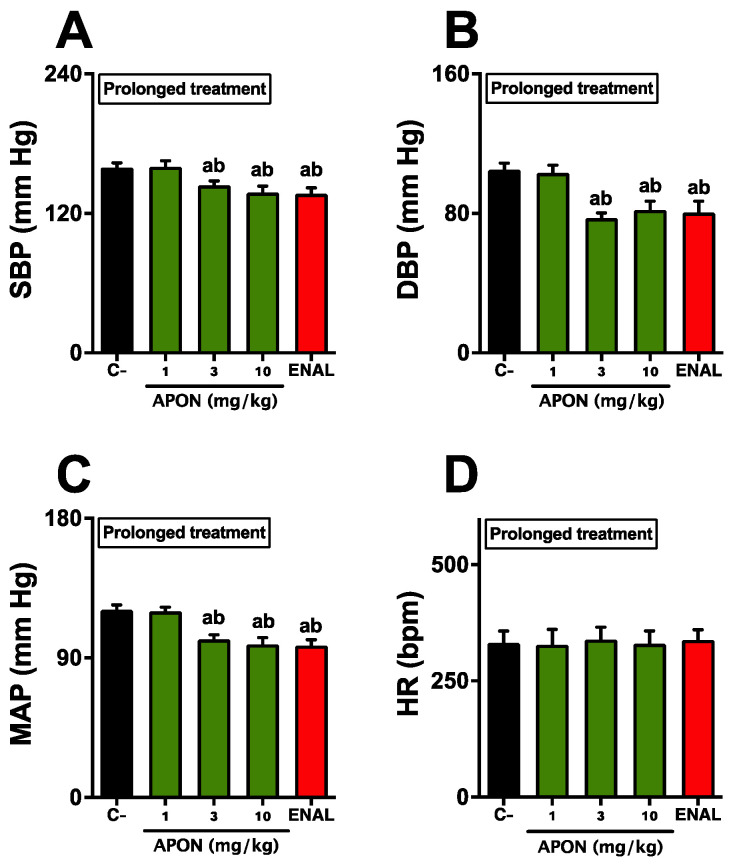
Sustained antihypertensive effects of Attalea phalerata oil-loaded nanocapsules (APON). APON was administered orally daily for 7 days. The systolic blood pressure (SBP; **A**), diastolic blood pressure (DBP; **B**), mean arterial pressure (MAP; **C**), and heart rate (HR; **D**) are displayed. C-, effect of vehicle (filtered water, 300 μL). The results are expressed as mean ± SEM (*n* = 6/group). ^a^ *p* < 0.05 when compared with respective control group; ^b^ *p* < 0.05 when compared with 1 mg/kg APON (ANOVA followed by Bonferroni post hoc test).

Seven days of oral administration of APON at doses of 3 and 10 mg/kg resulted in a significant decrease in blood pressure in all hypertensive rats. Basal measurements in control animals showed SBP, DBP, and MAP levels at 166.3 ± 4.7, 97.9 ± 4.2, and 120.3 ± 4.0 mmHg, respectively. However, in animals treated with 3 and 10 mg/kg of APON, the SBP, DBP, and MAP levels decreased to 144.1 ± 4.3, 75.9 ± 4.8, and 100.4 ± 4.4 mmHg, respectively (Figure 3A–C). It is important to note that heart rate did not show any significant changes with any of the treatments (Figure 3D). Additionally, SHR treated with enalapril also showed a significant decrease in blood pressure levels, comparable to the values seen in rats given 3 or 10 mg/kg of APON.

### 3.5. Effects of the Prostaglandin/Cyclic Adenosine Monophosphate Pathway on the Antihypertensive Effects of the APON

**Figure 4:**
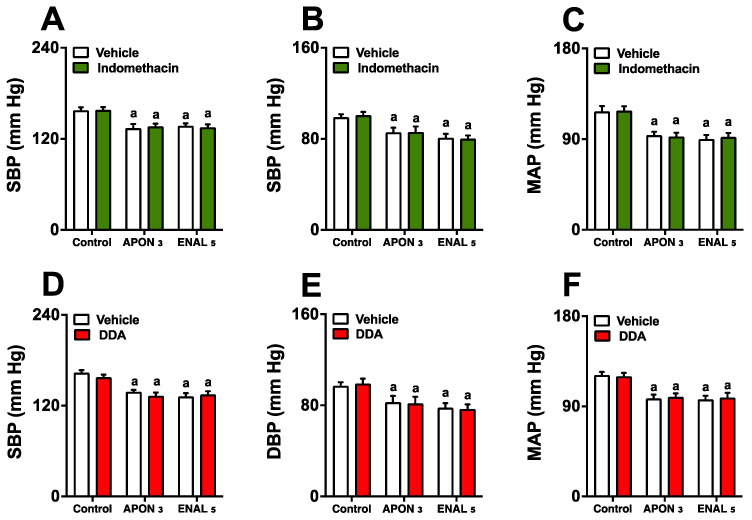
Absence of the involvement of the prostaglandin/cAMP pathway in the antihypertensive effect of *Attalea phalerata* oil-loaded nanocapsules (APON). The animals received APON (3 mg/kg, intraduodenally) in the presence and absence of an intraperitoneal injection of indomethacin (3 mg/kg) or DDA (0.1 mg/kg). The systolic blood pressure (SBP; **A**,**D**), diastolic blood pressure (DBP; **B**,**E**), mean arterial pressure (MAP; **C**,**F**) are displayed. ^a^ *p* < 0.05 when compared with respective control group (ANOVA followed by Bonferroni post hoc test). DDA, 2′,5′-dideoxyadenosine.

The decrease in blood pressure caused by APON was not affected when combined with indomethacin (Figure 4A–C) or adenylate cyclase inhibitor 2’,5’-dideoxyadenosine (DDA; Figure 4D–F). Furthermore, the use of indomethacin and DDA did not result in any changes in heart rate in any of the experimental groups.

### 3.6. Effects of the NO-cGMP-K^+^ Channel Pathways on the Antihypertensive Effects of the APON

**Figure 5:**
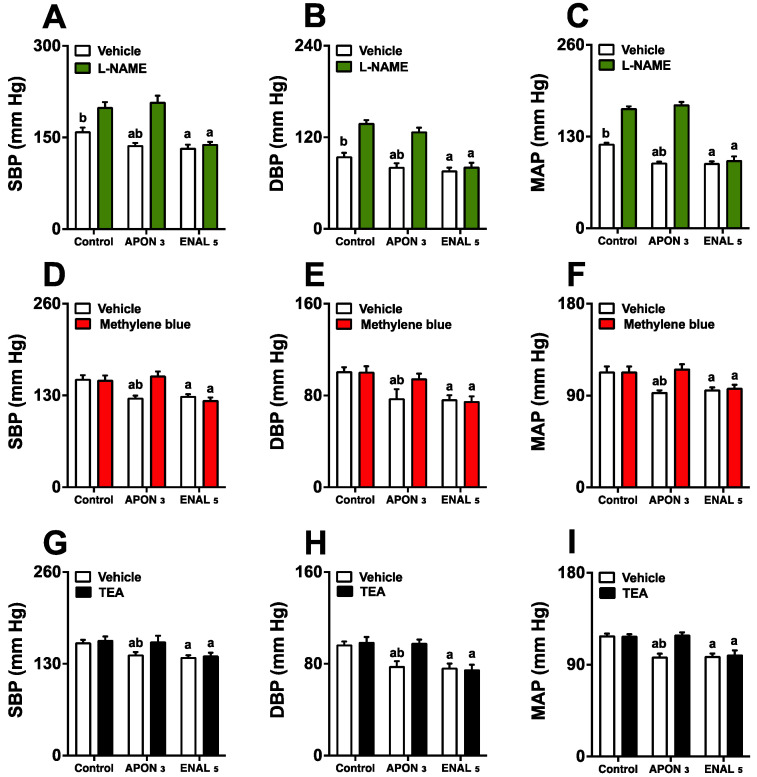
Involvement of the nitric oxide/cGMP pathway and K^+^ channels in the antihypertensive effect of Attalea phalerata oil-loaded nanocapsules (APON). The animals received APON (3 mg/kg, intraduodenally) in the presence and absence of a continuous infusion of L-NAME (7 mg/kg/min), a continuous infusion of methylene blue (150 nmol/kg/min), or an intraperitoneal injection of tetraethylammonium (400 μmol/kg). The systolic blood pressure (SBP; **A**,**D**,**G**), diastolic blood pressure (DBP; **B**,**E**,**H**), mean arterial pressure (MAP; **C**,**F**,**I**) are displayed. The results are expressed as mean ± SEM (*n* = 6/group). ^a^ *p* < 0.05 when compared with respective control group; ^b^ *p* < 0.05 when compared with respective inhibitor (ANOVA followed by Bonferroni post hoc test). L-NAME, Nω-Nitro-L-arginine methyl ester; TEA, tetraethylammonium.

The continuous administration of L-NAME resulted in a significant increase in systolic blood pressure (SBP) from 171.1 ± 5.1 mmHg to 205.6 ± 8.7 mmHg, diastolic blood pressure (DBP) from 98.5 ± 4.6 mmHg to 125.4 ± 5.2 mmHg, and mean arterial pressure (MAP) from 119.8 ± 4.7 mmHg to 154.7 ± 7.4 mmHg. L-NAME infusion impaired the ability of 3 mg/kg of APON to reduce SBP, DBP, and MAP, while only slightly affecting the effectiveness of enalapril (Figure 5A–C). Moreover, the administration of methylene blue (Figure 5D–F) or nonselective calcium-sensitive potassium channel blocker tetraethylammonium (TEA) (Figure 5G–I) abolished the antihypertensive effects of APON without changing the efficacy of enalapril.

## 4. Discussion

The oil extracted for this study is a non-viscous and translucent liquid with a yellowish hue, with the smell of ripe fruit. The extraction process revealed a yielding of 35.25% (*w*:*w*). The APO refractive index, relative density, acidity value, iodine value, and saponification value agree with those reported by Acácio et al. [8]. APO exhibited a fatty acid profile with dodecanoic acid (C_12:0_) as the most abundant (42.97%) saturated acid and oleic acid (C_18:1n9c_) as the prevalent unsaturated fatty acid (19.22%). APO shares physicochemical characteristics consistent with the results described in the literature [8,10]. This fact occurs because we use the same row material, methodology, and characterization methods. These properties play a crucial role as they can be used as quality control parameters to facilitate scalability for possible industrial production of the APO. Notably, beyond the research conducted by Acácio et al. [8] and our research, there are no existing studies focused on the use of APO. This lack of reference points underscores the need for further investigation, especially for potential commercial applications of this extractivist oil in cosmetics and pharmaceuticals.

The development of nanoencapsulated drugs based on ethnopharmacological knowledge provides a promising alternative for innovation in medicine [12]. This approach combines efficiency and cost-effectiveness [11,12]. Nanophytopharmaceuticals have the capability to address challenges associated with herbal products, including low solubility, limited bioavailability, and shelf-life concerns. The APON exhibited a pH of 5.14, which is typical of nanocapsules prepared using Kollicoat MAE 100P [13,14]. On the other hand, APON presented a particle size of 196.90 nm with excellent homogeneity in size, represented by the narrow peak base and a low polydispersity index (0.159). The APON exhibited a ζ-potential of −59.10 mV. The high modular value of ζ-potential allows the occurrence of strong particle–particle interaction, keeping nanoparticles in suspension, preventing aggregation, precipitation, and agglomeration processes, thus enhancing the APON kinetic stability [13,15].

The effect of *A. phalerata* fruit kernel oil on blood pressure has not been investigated until today. In our investigation, we explored the antihypertensive effects of APON in SHR, uncovering the molecular mechanisms involved in the cardiovascular effects. As a first step, we perform a screening of the antihypertensive activity after a single administration via the duodenum. This procedure avoids interaction with stomach food and allows for better standardization of absorption time and pharmacological response. Additionally, we also evaluated the effect after 7 days of oral administration, aiming to determine if the antihypertensive effect would be sustained. In both cases, we observed that doses of 3 and 10 mg of APON were able to induce a similar antihypertensive effect; therefore, we chose to use the lower dose (3 mg/kg) for investigative studies on the mechanism of molecular action.

In recent decades, there has been a focus on the role of endothelium-derived vasoactive mediators in the cardiovascular field [16]. The endothelium regulates vascular homeostasis by synthesizing and releasing substances that either constrict or relax blood vessels in response to chemical (internal or external) or physical stimuli, such as shear stress and pulsatile stretch. While the nitric oxide and prostacyclin produced by endothelial cells have a significant impact on controlling the tone of large arteries, endothelium-derived hyperpolarizing factors play a crucial role in smaller resistance arteries [17]. Our study demonstrated that the antihypertensive effects of APON were not affected neither by inhibiting cyclooxygenase with indomethacin, nor by the use of DDA, a selective adenylate cyclase inhibitor. On the other hand, the use of L-NAME, a nitric oxide synthase inhibitor, as well as methylene blue (a guanylyl cyclase inhibitor), abolished the antihypertensive response to APON. Therefore, it is reasonable to suggest that APON-induced blood pressure reduction is directly influenced by the release of endothelial nitric oxide.

To investigate the role of downstream pathways in the nitric oxide-dependent activity of APON, we used a traditional K^+^ channel blocker, tetraethylammonium, which completely reversed the antihypertensive effects of APON. These results suggest that the activation of potassium channels is an important step for APON-induced antihypertensive effects. Given that the downstream targets of the nitric oxide pathway in blood vessels involve the opening of K^+^ channels [18], it is reasonable to propose that the NO-cGMP-K^+^ channel pathway contributes to the endothelium-dependent effects of APON in the SHR.

Despite the valuable insights gained from our study, there are certain limitations that need to be considered. We recognize that our investigation did not delve into the specific mechanisms that may be responsible for modulating NO production or inactivation by APON. Due to the known interaction between antioxidants and NO availability, additional research is needed to clarify the exact mechanisms by which APON affects NO signaling and the implications it has on vascular function.

## 5. Conclusion

Our study demonstrated a significant antihypertensive effect of *Attalea phalerata* oil-loaded nanocapsules in spontaneously hypertensive rats (SHR) following both acute and prolonged (7-day) administration. This effect appears to be dependent on the function of the vascular endothelium and involves the NO-cGMP-K^+^ channel pathway. By elucidating this pathway’s involvement, our findings contribute to a deeper mechanistic understanding of APON’s pharmacological effect. This work reports, for the first time, the potential therapeutic value of APON for hypertension management, along with the mechanistic insights into its cardiovascular effects. This research opens a new avenue for future investigations aimed at definitively establishing APON’s therapeutic utility in managing cardiovascular disease.

## Figures and Tables

**Table 1:** Fatty acid profile of the Acurí kernel oil.

Chain Length	Fatty Acid	Relative Quantity (%)
C_8:0_	Octanoic acid	8.91 ± 0.31
C_10:0_	Decanoic acid	7.67 ± 0.74
C_12:0_	Dodecanoic acid	42.97 ± 1.55
C_14:0_	Tetradecanoic acid	10.09 ± 0.37
C_16:0_	Hexadecanoic acid	6.02 ± 0.49
C_18:0_	Octadecanoic acid	2.42 ± 0.08
C_18:1n9c_	9-octadecenoid acid	19.22 ± 0.85
C_18:2n6c_	9,12-octadecadienoid acid	2.88 ± 0.14
	Total	99.18 ± 0.49

The analysis was made in triplicate. Saturated fatty acids: 77.08 ± 0.59%, Unsaturated fatty acids 22.10 ± 0.26%.

## Data Availability

The raw data supporting the conclusions of this article will be made available by the authors on request.
